# Role of apoptotic, autophagic and senescence pathways in minor salivary gland adenoid cystic carcinoma

**DOI:** 10.1186/s13000-019-0796-2

**Published:** 2019-02-08

**Authors:** João Augusto Vianna Goulart-Filho, Victor Angelo Martins Montalli, Fabrício Passador-Santos, Ney Soares de Araújo, Vera Cavalcanti de Araújo

**Affiliations:** Department of Oral Pathology, São Leopoldo Mandic Research Institute, Rua José Rocha Junqueira, 13, CEP, Campinas, SP 13045-610 Brazil

**Keywords:** Adenoid cystic carcinoma, Angiogenesis, Apoptosis, Autophagy, Senescence

## Abstract

**Background:**

Adenoid cystic carcinoma (ACC) is a salivary gland malignancy with poor long-term survival, which warrants studies aimed at clarifying the pathogenesis of this disease in order to widen the scope of therapeutic options currently available. Alterations in regulatory mechanisms relating to vascular support, cell death and autophagy are important pathways for tumor growth in cancer. Thus, the present study aimed to access vascular supply, apoptosis, autophagy and cell senescence in ACC of minor salivary glands.

**Methods:**

We analyzed 25 cases of minor salivary gland ACC by immunohistochemistry using anti-CD34, anti-CD105, anti-D2–40, anti-Bax, anti-Bcl-2, anti-Beclin-1, anti-LC3B, anti-p21 and anti-p16.

**Results:**

Microvessel density was low and based on anti-CD34, anti-CD105 and anti-D2–40 immunostaining. There was positivity for anti-CD34, anti-Bcl-2, anti-Beclin, anti-LC3B and anti-p21 and a positive correlation between Bcl-2 and Beclin (*p* = 0.014).

**Conclusions:**

Our results showed that ACC does not depend on neo-angiogenesis and is probably associated to anti-apoptotic, autophagic and anti-senescence events.

## Background

Adenoid cystic carcinoma (ACC) represents 1% of all head and neck cancers and 10–15% of all salivary gland tumours and is characterized by indolent growth, perineural invasion and multiple local recurrences. In contrast to rare regional lymphatic metastasis, hematogenous metastases occur particularly to the lungs, bones and liver [1–3]. Surgical resection followed by radiotherapy is the treatment of choice, but the relentless nature of tumour growth and high potential for local recurrences usually makes treatment difficult, mutilating and non-responsive to chemotherapy, contributing to a poor prognosis and making many authors consider ACC as a “clinically high-grade” neoplasm [4–6]. Microscopically, ACC is composed by luminal and myoepithelial cells and may present three distinct patterns: cribriform, tubular and solid, the latter showing the worst prognosis and poorest survival [3, 6, 7].

This study aims to evaluate microvascularization (CD34 and CD105), apoptosis (Bcl-2 and Bax), autophagy (Beclin and LC3B) and senescence (p21 and p16) in minor salivary gland ACC.

## Methods

Twenty-five formalin-fixed paraffin-embedded samples of ACC derived from minor salivary glands were obtained from the archives of the Oral Pathology Laboratory of São Leopoldo Mandic Institute and Research Center. All cases underwent immunohistochemical staining for the following antibodies: anti-CD34 and anti-CD105 for blood vessels; anti-D2–40 for lymphatic vessels; anti-Bcl-2 and anti-Bax for apoptosis; anti-Beclin and anti-LCB3 for autophagy; and anti-p21 and anti-p16 for senescence. This study was approved by the Research Ethics Committee of the Sao Leopoldo Mandic Research Institute (protocol number: 66460817.6.0000.5374).

Five-micrometer sections were dewaxed, rehydrated and endogenous peroxidase activity was quenched by immersing slides in 3% hydrogen peroxide. Antigen retrieval (AR) was achieved by immersing the slides in boiling citrate buffer (pH: 6.0) for anti-CD34, anti-Bcl-2, anti-Bax, anti-Beclin, anti-LC3B, anti-p21 and anti-p16. For anti-CD105 staining, AR was performed using 4% pepsin at 37 °C, whilst for anti-D2–40, Tris-EDTA was used for 30 min. Only the sections for anti-CD105 staining were incubated at 37 °C with serum-free protein blocking solution (code × 0909, Dako, SA, and Denmark) for 10 min before incubating the antibody. Subsequently, the sections were incubated with the primary antibody for 60 min (anti-CD34, anti-Bax, anti-Bcl-2, anti-Beclin, anti-LC3B, anti-P21) or overnight (anti-CD105, anti-D2–40, anti-p16) at 4 °C followed by En Vision polymer HRP and Envision+ (code K1491, DAKO, SA, Denmark) for 1 h at 37 °C. The sections were stained for 5 min at 37 °C with 3,3′- diaminobenzidine tetrahydrochloride (DAB) and counter-stained with Harris’s hematoxylin. A negative control was obtained by omitting primary antibody, when no staining was observed.

As incisional biopsies provide a limited amount of tissue for analysis, some paraffin blocks did not contain sufficient material for all immunohistochemistry reactions, hence the different numbers reported in results.

To verify possible differences in immuno-expression across the histological patterns of ACC, we divided the samples into solid and non-solid (which includes the cribriform and tubular subtypes) as proposed by van Weert et al. [2] Ten sections per tumor were obtained and analyzed to exclude the possibility of tumor heterogeneity.

Immunostaining for CD34, CD105 and D2–40 was interpreted by two experienced pathologists (VCA and JAVGF) using a double-headed microscope. Vascularization was accessed on the highest cellular areas through images obtained from 5 fields (hotspots) per case (40X objective, 0.44 mm field diameter) using a digital camera (Infinity 1, Canada) coupled to an Olympus CX30 microscope. The images were evaluated on Imagelab analysis software (version 2.4), which allowed manual segmentation of target vessels. Microvascular density for blood (MVD) and lymphatic vessels (LVD) were expressed as the mean value of the microvessels counted. Only vessels in the stroma around the tumour epithelial islands were counted. Tissue fragments located on the margins of the tumor and not belonging to the stroma were defined as peritumoral areas. There was no restriction on the size of countable microvessels. Vessels presenting muscle walls and necrotic areas were excluded.

Immunostained sections for Bcl-2, Bax, Beclin, LC3B, p21 and p16 were evaluated qualitatively and semi-quantitatively. Qualitative analysis was based on positive cells throughout the tissue section, observing the absent, weak or strong staining for each marker as well as its distribution throughout the specimen. The relative number of positively-stained cells was considered in relation to all neoplastic cells observed in each section. Based on percentage of positive cells, cases were scored as: 0 (less than 5% positive cells), 1 (5–50% positive cells) and 2 (higher than 50% positive cells).

The ratio Bax and Bcl-2 staining was obtained by the coefficient between Bax/Bcl-2 scores for each case. Values close to zero indicate absence of apoptosis whereas those close to 2 indicate apoptotic activity.

The CD34 and CD105 immunoexpression was analyzed by *t-Student* test. Bcl-2, Bax, Beclin, LC3B, p21 and p16 scores were analyzed using the *Mann-Whitney* test. The correlation between the expression of different markers was measured using *Pearson’s r* test at a significance level of 5%. Statistical calculations were performed on GraphPad Prism 6.

## Results

**Fig. 1:**
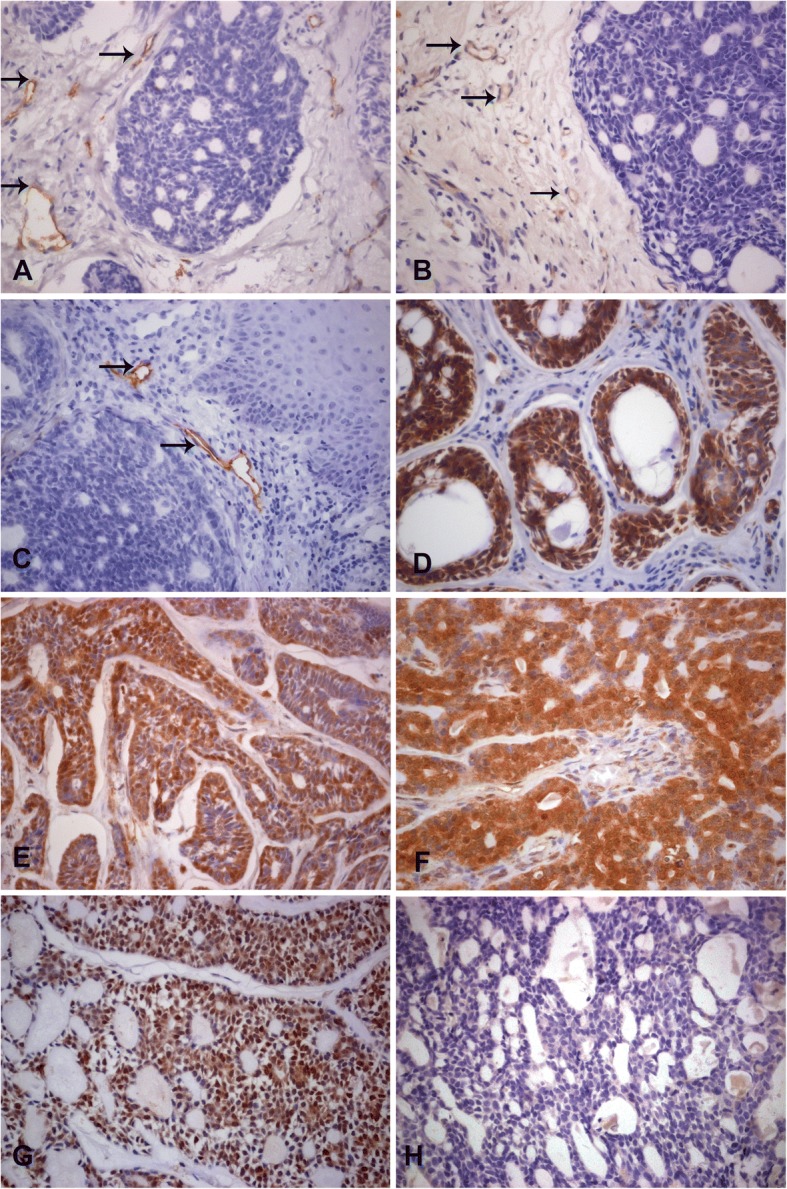
Immunohistochemistry panel for adenoid cystic carcinoma (ACC) showing expression of CD34 (arrows) in the peritumoral stroma (**a**), unusual CD105 (arrows) positive vessels in peritumoral stroma of rare cases (**b**), infrequent positivity for D2–40 (arrows) in peritumoral stroma (**c**), strong Bcl-2 expression in neoplastic cells (**d**), strong Beclin expression in neoplastic cells (**e**), an area with intense positivity for LC3B (**f**), strong p21 positivity in neoplastic cells (**g**), and negative expression for p16 in neoplastic cells (**h**)

The age at diagnosis ranged from 19 to 88 years in our sample, with mean age of 51,76 years (SD = 15,98), being most frequent in females (15 cases; 60%) than in males (10 cases; 40%) with a rate of 1,5:1. Palate was the most affected site (10 cases; 40%), followed by maxilla (5 cases; 20%), floor of the mouth (5 cases; 20%), buccal mucosa (3 cases; 12%) an upper lip (2 cases; 8%). Clinical data are summarized in Table 1. Regarding histological subtype, 15 cases of non-solid (10 of cribriform and 5 of tubular) and 10 cases of solid were observed. When vasculature was evaluated, immunostaining for CD34 detected 3.86 ± 0.42 vessels in general, while CD105 detected a very low number of new vessels (0.39± 0.26) at peritumoral site (Fig. 1a and Fig. 1b).

**Table 1:** Clinical features of the ACC evaluated

Case	Age	Gender	Site
1	F	58	Palate
2	F	43	Buccal mucosa
3	M	56	Upper lip
4	M	47	Floor of the mouth
5	F	42	Maxilla
6	F	34	Floor of the mouth
7	F	37	Floor of the mouth
8	F	47	Buccal mucosa
9	F	73	Upper lip
10	M	55	Maxilla
11	F	65	Palate
12	F	38	Palate
13	F	46	Floor of the mouth
14	M	36	Palate
15	M	62	Maxilla
16	M	88	Palate
17	F	63	Buccal mucosa
18	M	69	Palate
19	F	48	Palate
20	F	49	Palate
21	F	57	Floor of the mouth
22	F	–	Palate
23	M	19	Palate
24	F	60	Maxilla
25	F	75	Maxilla

Positivity for lymphatic vessels was cytoplasmic (Fig. 1c) and evidenced in a very small number of vessels restricted to peritumoral areas.

Strong membrane and cytoplasmic expression of Bcl-2 (score 2) was noted in 19 cases (76%) (Fig. 1d). Weak positivity (score 1) was noted in 4 cases (16%) and 2 cases were negative (8%). There was no significant difference between Bcl-2 expression in solid and non-solid ACCs (*p* = 0.53).

Bax expression was remarkably negative and rarely granular and cytoplasmic. Under the established score, 24 cases scored 0 (96%), 1 case scored 1 (4%) and no case scored 2. There was no difference between solid and non-solid subtypes (*p* = 0.32). Distribution data for Bax and Bcl-2 are shown in Table 2.

**Table 2:** Distribution data for Bcl-2, Bax, Beclin, LC3B, p21 and p16 used for immunohistochemistry according to established scores and *p* value for difference between solid and non-solid ACCs

Score	Bcl-2		Bax		Beclin		LC3B		p21		p16		CD34 (%)	CD105 (%)
	S	NS	S	NS	S	NS	S	NS	S	NS	S	NS	Mean (SD)	Mean (SD)
	n (%)	n (%)	n (%)	n (%)	n (%)	n (%)	n (%)	n (%)	n (%)	n (%)	n (%)	n (%)		
0	0 (0,0)	2 (11.8)	7 (87.5)	17 (100)	0 (0,0)	0 (0,0)	3 (37.5)	8 (47.1)	0 (0,0)	4 (23.5)	8 (100)	16 (100)		
1	3 (37.5)	1 (5.9)	1 (12.5)	0 (0,0)	3 (37.5)	1 (5.9)	1 (12.5)	3 (17.6)	1 (14.3)	2 (11.8)	0 (0,0)	0 (0,0)	3.8 (0.4)	0.39 (0.2)
2	5 (62.5)	14 (82.4)	0 (0,0)	0 (0,0)	5 (62.5)	16 (94.1)	4 (50)	6 (35.3)	6 (85.7)	11 (64.7)	0 (0,0)	0 (0,0)		
*p*	• 0.53		• 0.32		• 0.08		• 0.64		• 0.26		–		■ 0.0001	

Legend: 0 = absent; 1 = weak; 2 = strong staining

• *= Mann-Whitney* test for S (solid) and NS (non solid) minor salivar ACC

■ *=* Student t test

*Pearson correlation Bcl-2 and Beclin = p = 0.014; r = 0.483*

Bax-Bcl-2 ratio for most cases (*n* = 24) was 0 and 0.5 for 1 case, indicating that virtually no apoptosis was taking place.

Membrane and cytoplasmic Beclin expression revealed strong positivity in most cases (Fig. 1e), where 21 cases (84%) scored 2 and 4 cases scored 1 (16%) with no significant difference in Beclin expression between solid and non-solid ACC (*p* = 0.08).

Cytoplasmic immunostaining for LC3B was negative in 11 (44%) cases, weak (score 1) in 4 cases (16%) and strong (score 2) in 10 cases (40%) (Fig. 1f). No significant difference was noted between solid and non-solid ACC (*p* = 0.64). Data distribution for Beclin and LC3B are shown in Table 2.

Nuclear staining for p21 was observed in 24 cases (Fig. 1g), where four (16,6%) scored 0, three scored 1 (12,5%) and seventeen scored 2 (70,9%). No significant difference was noted between solid and non-solid ACCs (*p* = 0.26). p16 expression was negative in all cases (Fig. 1h). Data distribution for p21 and p16 are shown in Table 2.

A statistically positive correlation was found between the expression of Bcl-2 and Beclin (*p* = 0.014; r^2^ = 0.483).

## Discussion

ACC is the second commonest malignancy of minor salivary glands and its natural history includes high risk of late distant metastasis to lungs, liver and bone [4, 7]. Although survival is relatively high at 5 years, ACC mortality increases significantly between 5 and 20 years [8].

Our clinical data highlight the palate as the commonest intraoral site for ACC with a peak incidence after the sixth decade of life (51,76±15,98 years) as well as a slight predilection for women, as previously described [7].

Cribriform and tubular histological patterns are generally associated with longer survival rates, lower metastasis and therefore better prognosis than seen in the solid variant [3]. In our series, cribriform and solid were the most common patterns and showed a similar distribution across the sample.

Recurrent translocations t(6;9)(q22–23;p23–24) have been demonstrated in 30–50% salivary ACCs, causing formation of MYB-NFIB fusion oncogene, resulting in loss of MYB repression, which induces transcriptional activation of MYB target genes associated with cell cycle control, apoptosis, cell growth, angiogenesis and cell adhesion [8].

CD34 positivity in preexisting blood vessels within the tumour stroma but negativity or very scarce CD105 expression indicates that ACC may use another source of nutrients to sustain its growth, as supported by previous studies [9]. Angiogenesis is upregulated early in invasive cancers and provides nutrients and oxygen as well as drainage for metabolic byproducts [9], which does not seem to occur in ACC.

D2–40 staining was practically absent in our series. When present, it was restricted to the periphery of the tumour as small and constricted vessels that probably were pre-existing lymphatic vessels. This finding is in accordance with Fujita et al. [10] and indicates that lymphangiogenesis does not occur in ACC and is compatible with rare lymph node metastasis, which is typical to this malignancy [1, 5].

Unbalanced Bax/Bcl-2 ratio suggested that anti-apoptotic mechanisms are critical for tumour growth in ACC. Apoptosis is present in homeostatic regulation of cell populations, cell stress responses and serves as a natural barrier to cancer development [9, 11]. On the other hand, deregulation of apoptosis in favor of anti-apoptotic events contributes to accumulation of DNA-damaged cells, tumour progression and is probably involved with the indolent course and chemotherapy resistance commonly seen in ACC [11–13].

Recent studies demonstrated that overexpression of pro-survival members of the Bcl-2 family can also promote cell migration, invasion and metastasis in a wide spectrum of malignancies via stimulation of Reactive Oxygen Species (ROS) that trigger activation of diverse sets of signaling pathways, such as kinases (PI3K, MAPKs), transcription factors (AP-1, Sp1), cell surface receptors (EGFR, FGFR) and matrix-degrading enzymes (MMP-2 and MMP-9) [14]. Strong evidence suggests that these Bcl-2 functions are mediated by an inhibitory effect over pro-apoptotic proteins, such as Bax. By binding to Bax, Bcl-2 prevents interaction of Bax with the protein complex-I in the intermembrane space of mitochondria and blocks the inhibitory effect of Bax on ROS production [14].

Protein Bcl-2 also exerts a regulatory effect on autophagy. In human cancers, dysregulation of autophagy may act as an adaptive mechanism that protects tumor cells from events that may trigger apoptosis [15].

Autophagy is a catabolic mechanism with homeostatic functions present in low basal levels under physiological conditions, mainly implicated in intracellular pathways for degradation and recycling of proteins and organelles. It is profoundly induced by stress and nutrient starvation and plays a cytoprotective or an apoptosis-inducing role in critically dmaged cells that cannot be rescued [16].

The strong positivity for Beclin and heterogeneous positivity for LC3B suggest that instead of a vascular support, autophagy may play an important role in ACC pathogenesis as well as in the development of chemotherapy resistance [17]. The similar expression of Beclin in non-solid and solid ACCs in our series differs from the findings reported by Jiang et al. [18], where loss of Beclin expression in solid tumours was an indicator of poor prognosis.

Up-regulation of autophagy in cancer cells is related to sustained metabolism and survival by degrading macromolecules in lysosomes fueling mitochondria with substrate, preventing energy crisis and fatal nucleotide pool depletion in nutrient starvation and hypoxic tumour regions [16, 19]. Recent evidence indicates that autophagy also acts preventing p53 activation, growth arrest, apoptosis, senescence and activation of the immune response [16, 20].

There is cross-talking between apoptosis and autophagy, where suppression of apoptosis induces autophagy and vice-versa [13, 10, 19, 20]. Our results support this assumption, since we observed a positive correlation for simultaneous strong expression for Bcl-2 and Beclin.

Beclin 1 regulates membrane trafficking and it is essential for localization of autophagic proteins to a pre-autophagosomal structure during the autophagy nucleation phase. It is considered an important convergence point of autophagy and apoptosis because this protein has a BH3-only domain that binds to Bcl-2 in the endoplasmic reticulum surface. Under physiologic conditions, the Beclin 1/Bcl-2 complex inhibits autophagy [13, 15]. However, Beclin 1 does not have the ability to neutralize the anti-apoptotic function of Bcl-2. Mutations to either the BH3-only domain within Beclin 1 as well as in its receptor domain within Bcl-2, disrupt the Beclin 1/Bcl-2 complex and result in stimulation of autophagy [21].

Some tumour suppressor pathways involved in cell cycle control, such as cyclin-dependent kinase inhibitors p21 and p16, are also deregulated in human cancers [22]. Despite not being specific markers of senescence, p21 plays a transient inhibitory role on pRb while p16 ensures a state of permanent pRb hypophosphorilation [12]. We noted a predominant strong and intense expression of p21 contrasting with complete negativity for p16. The absence of p16 is in agreement with some previous studies and may be associated with a possible mutation, deletion or even methylation of the p16 gene, leading to an unrestricted progression from G1 to S phase [23–25]. Nevertheless, the results on p16 expression in ACCs are conflicting in the literature, with some studies showing positivity for p16 [25, 26], while others reported expression only in high-grade tumours [27]. Increased p21 immunostaining may indicate a transient p53-dependent inhibitory activity, insufficient to maintain cell cycle arrest in malignant cells due to deficient p16 expression, avoiding cell death by senescence and favoring tumour growth, as seen in other malignancies [22, 28, 29].

Our results showed no difference between solid and non-solid ACCs, suggesting that similar pathways sustain tumour growth in both histological subtypes. In this context, the worse prognosis for solid subtype may be related to different factors, possibly those associated with metastasis.

## Conclusions

In conclusion, ACC has its growth based on autophagy, anti-apoptotic and anti-senescense mechanisms, as recently reported for Polymorphous adenocarcinoma [12], suggesting common disrupted pathways for several slow-growing salivary gland malignancies.

## Abbreviations

ACC: Adenoid cystic carcinoma
